# Women empowerment and hypertension in Nepal: a nationally representative survey analysis

**DOI:** 10.1057/s41271-025-00593-7

**Published:** 2025-08-13

**Authors:** Md. Shajedur Rahman Shawon, Mohammad Rifat Rahman, Tanij Fahima, Ferdousy Jannat, Fariha Binte Hossain

**Affiliations:** 1 Centre for Big Data Research in Health, UNSW Sydney, Level 2, AGSM Building (G27), Sydney, NSW 2052 Australia; 2 Department of Banking & Insurance, Faculty of Business Administration, University of Chittagong, Chattogram, Bangladesh; 3 Mukti Cox’sBazar, Cox’sBazar, Bangladesh; 4 Independent University, Dhaka, Bangladesh; 5 School of Population Health, University of New South Wales, Sydney, NSW 2033 Australia

**Keywords:** Women empowerment, Hypertension prevalence, Hypertension awareness, Hypertension treatment, Nepal

## Abstract

This study investigates the roles of women empowerment on the prevalence, awareness, and treatment of hypertension among ever-married Nepalese women aged 15–49 years using 2016 Nepal Demographic and Health Survey data. We assessed women empowerment using the Survey-based Women emPowERment (SWPER) index in the domains of ‘social independence’, ‘decision-making’, and ‘attitudes towards violence’. We assessed associations of domains of women empowerment with hypertension using multiple logistic regression models. Among 4919 women, 568 (12%) had hypertension, 35% were aware of their condition, and 14% were taking anti-hypertensive medication. Majority (72%) of women had high empowerment in ‘‘attitude to violence’ domain, 14%—in ‘decision-making’ domain, and 25%—in the ‘social independence’ domain; the latter group was also less likely to suffer from hypertension (aOR = 0.68, 95% CI 0.49–0.94). Our findings suggest the need to integrate empowerment initiatives into strategies to prevent hypertension among women in Nepal and similar low- and middle-income settings.

**Supplementary Information:**

The online version contains supplementary material available at 10.1057/s41271-025-00593-7.

## Key messages

- High empowerment in the ‘social independence’ domain is linked to a lower risk of hypertension among women in Nepal.
- Awareness and treatment of hypertension among Nepalese women remain low, with only 35% aware of their condition and 14% receiving treatment.
- Empowerment in ‘attitudes towards violence’ and ‘decision-making’ did not show a significant impact on hypertension prevalence or management.
- The study highlights the need for more targeted efforts to improve health awareness and healthcare access for women with low empowerment.

## Introduction

Hypertension, or high blood pressure, is a significant global health concern affecting approximately 1.28 billion adults aged 30–79 years, of which two-thirds reside in low- and middle-income countries (LMICs) [1]. It is a major contributor to morbidity and mortality associated with cardiovascular disease. South Asian regions, including Nepal, have reported some of the lowest rates of hypertension detection, treatment, and control [2]. Nepal is currently experiencing an epidemiological transition with a shift in disease burden from communicable diseases to non-communicable diseases (NCDs) and previous studies reported that nearly one in four adults in Nepal have hypertension [3–6]. A wide array of socio-economic factors including but not limited to socioeconomic status, education, employment, and access to healthcare can influence hypertension risk and its management [7]. Thus, addressing social determinants of health is critical for comprehensive and effective strategies aimed at preventing and managing hypertension.

Launched in 2015, the Sustainable Development Goals (SDGs) prioritize the achievement of gender equality and empowerment for all women and girls (SDG 5) [8]. Women empowerment significantly enables them to maintain their health and access necessary health resources [9]. Such empowerment enhances their ability to navigate social and economic environments effectively, leading to improved health behaviors and more informed health-related decisions, including when to seek preventive or curative care. Previous studies have confirmed the positive impact of women’s empowerment on health outcomes, such as increased use of modern contraception, access to antenatal care, skilled birth attendance, and vaccinations for children [10–12]. The 2021 Beijing Declaration of the World Health Organization (WHO) highlighted the reduction of noncommunicable diseases such as hypertension among women as one of the priority areas [13]. However, relationship between women empowerment and hypertension prevalence and management remains unexplored.

Women empowerment is inherently complex and multi-dimensional, and therefore difficult to measure, particularly in the context of LMICs [14]. However, researchers have recently developed the survey-based Women’s emPowERment (SWPER) index, an innovative instrument to measure women empowerment, using data from the Demographic and Health Surveys (DHS) [15, 16]. The SWPER index’s showed high external validity and robustness when compared with two well-recognized indices: the Gender Development Index (GDI) and the Gender Inequality Index (GII) [17].

Although Nepal is committed to achieving women’s empowerment and gender equality in line with SDG-5, there remains a disparity and inequality between men and women [18]. Considering the critical importance of hypertension as an important contributor to cardiovascular disease, and the essential societal role women play, a comprehensive understanding of women empowerment on hypertension prevalence, awareness, and treatment is necessary. Using recent DHS data from Nepal, this study aims (i) to measure levels of women empowerment according to the SWPER index among ever-married women aged 15–49 years, (ii) to estimate the prevalence of hypertension and the percentages of awareness and treatment of hypertension among those with hypertension, and (iii) to quantify the associations of women empowerment with hypertension prevalence, awareness, and treatment.

## Data and methods

### Datasets and study design

This cross-sectional study used secondary data from the 2016 Nepal Demographic and Health Surveys (2016 NDHS) [19], which included both blood pressure measurements and information needed to calculate the SWPER index [16]. The 2016 NDHS was executed by New ERA under the Ministry of Health of Nepal, with funding from the United States Agency for International Development (USAID) and technical assistance from ICF through The DHS Program, a USAID-funded project. The dataset, anonymized to protect privacy, were accessed from the DHS website [20] after submitting a research proposal, as per their guidelines.

The sampling frame for the 2016 NDHS was an updated version from the 2011 National Population and Housing Census (NPHC), provided by the Central Bureau of Statistics (CBS) [19]. This sampling frame provides details on ward location, residence type (urban or rural), projected number of residential households, and estimated population. The 2016 NDHS employed a two-stage selection process in rural areas and a three-stage process in urban areas. Rural wards were selected as primary sampling units (PSUs), followed by the selection of households from these PSUs. In urban areas, wards served as PSUs, one EA (enumeration area) was selected from each PSU, and households were chosen from these EAs. During the first stage, 383 wards were selected, with the probability of selection proportional to ward size, which is determined by the number of residential households. For large urban wards, a second stage involved randomly selecting one EA from each sample ward. A household listing operation was performed in all selected sampling clusters (rural wards or urban EAs), creating a list that served as the sampling frame for household selection in the subsequent stage. To reduce the task of household listing in clusters containing more than 200 households, such clusters were segmented, and only one segment was chosen for the survey, with the probability proportional to segment size. Finally, 30 households per cluster were selected systematically, with an equal chance of selection from the newly created household list. Further details of the sampling process is given in the DHS report [19]. Survey interviews were conducted exclusively in pre-selected households, and to avoid bias, no replacements or alterations were permitted. All women aged 15–49 who were permanent residents of the selected households were eligible for interviews, and for blood pressure measurements, with a response rate of 98% [19]. This analysis, however, is limited to currently married women who also had measured blood pressure data and the necessary information to calculate the SWPER index (38% of all eligible women).

The 2016 NDHS received ethical approval from the Nepal Research Council and the ICF Macro Institutional Review Board located in Calverton, Maryland, USA. Before proceeding with the interview, each participant gave their informed written consent [19].

### The survey-based women emPowERment (SWPER) index

The Survey-based Women emPowERment (SWPER) index was created through a method that employed key questions relating to women’s empowerment from Demographic and Health Surveys (DHS) [15, 16]. Higher scores were allotted to those responses that signified a higher level of empowerment (refer to Supplementary Table S1—for details about the questions and their corresponding scores). We limited the scope of the analysis to women in partnerships, given the nature of specific questions. For women without childbirth history, we used single hot-deck imputation method to infer the age of first birth [16].

The SWPER index encapsulates three primary domains: ‘attitude towards violence’, ‘social independence’, and ‘decision-making’. The first domain explores women’s attitudes towards the justification of domestic violence in different situations. The second domain, ‘social independence’, assesses a range of factors including a woman’s education, frequency of media interactions such as reading newspapers or magazines, and the age of major life events, like the first childbirth and cohabitation. The disparities in age and education between partners are also considered in this domain, albeit with lower weightings. The third domain, ‘decision-making’, evaluates the extent of a woman’s involvement in key household decisions, serving as a reflection of her autonomy within the home [15, 16].

To formulate the SWPER index, we applied a principal component analysis (PCA) on these 14 elements. Each item was then weighted to reflect its unique contribution to the total empowerment score. To maintain global relevance and comparability, we standardized the scores based on the global mean for LMICs and the standard deviation from a previous study [16]. These standardized scores were then divided into three categories—low, medium, and high levels of empowerment, guided by the standard cut-off points suggested by previous research that roughly splits global scores into tertiles [16].

### Outcome variables

We explored three aspects of hypertension: prevalence, awareness among those diagnosed with hypertension, and treatment rates among those aware of their hypertensive status. Blood pressure readings were taken three times from consenting women aged 15 and above, using UA-767F/FAC (A&D Medical) blood pressure monitors, with an interval of 5 min or more between each reading. The average of the last two readings, for both systolic and diastolic pressures, was used to determine hypertension. According to the guidelines set by WHO and the International Society of Hypertension [21], a respondent was defined as hypertensive if they had an average systolic blood pressure of 140 mmHg or more, OR an average diastolic blood pressure of 90 mmHg or more. Furthermore, respondents who reported that they were currently using any antihypertensive medication were also classified as hypertensive.

The term ‘awareness of hypertension’ was defined as those who had been previously diagnosed by a healthcare professional. This was confirmed by asking if they had ever been informed about having high blood pressure or hypertension by a doctor or other healthcare worker. As for the treatment of hypertension, any respondent classified as hypertensive and reported taking antihypertensive medication on the day of the survey was classified as ‘treated’.

### Covariates

We included various sociodemographic characteristics in this study, including survey year, age, place of residence, administrative province (Koshi, Madhesh, Bagmati, Gandaki, Lumbini, Karnali, Sudurpashchim), highest educational level of women and household wealth index. Age was categorized into 15–29 years, 30–39 years, and 40–49 years. The definitions of rural and urban residences were guided by country-specific parameters. The socioeconomic status (SES) of the household was derived from the 2016 NDHS household wealth index, which was calculated using principal components analysis based on quantity and variety of consumer goods they own and their housing characteristics, such as source of drinking water, toilet facilities, and flooring materials. We then assigned wealth index values to each household member and divided the population into national wealth quintiles, each containing 20% of the population, from poorest (Q1) to richest (Q5) [19].

### Statistical analysis

All analyses were performed in accordance with the DHS guide to analysis [22], utilizing Stata v16.1 software and taking into account the complex survey design with Stata’s “svy” command. We used descriptive statistics to estimate proportions for categorical variables and means and standard deviations for continuous variables in our sample. Chi-square tests was used to investigate the bivariate relationships between Women empowerment status, sociodemographic characteristics, and hypertension variables. Furthermore, we estimated the prevalence, awareness, and treatment of hypertension based on various sociodemographic factors and SWPER domains.

We utilised multiple logistic regressions to investigate the associations of the three SWPER domains with hypertension prevalence, awareness, and treatment. The adjusted odds ratios (aORs) with 95% confidence intervals (CIs) were estimated, with all covariates simultaneously entered into the multiple regression model.

We considered an alpha level (α) of 0.05 as the cut-off for statistical significance and all statistical tests were two-sided.

## Results

Our analysis included a total of 4919 women from the 2016 NDHS. Table 1 illustrates the sociodemographic characteristics of the women included in the study. The average age of the participants was 31.59 years (SD = 8.81) with a considerable majority (63.4%) residing in urban areas. Geographical distribution shows participants spread across all provinces, from Bagmati (12.0%) to Madhesh (13.4%). The levels of education varied substantially, with 41.0% of the women having no education and 11.4% achieving higher education.

**Table 1:** Sociodemographic characteristics of included participants

	Number (%)
No. of participants	4919
Mean age of women in years (SD)	31.59 (8.81)
Age groups	
15–29 years	2206 (44.8)
30–39 years	1609 (32.7)
40–49 years	1104 (22.4)
Area of residence	
Urban	3119 (63.4)
Rural	1800 (36.6)
Province	
Koshi	689 (14.0)
Madhesh	889 (18.1)
Bagmati	591 (12.0)
Gandaki	610 (12.4)
Lumbini	772 (15.7)
Karnali	711 (14.5)
Sudurpashchim	657 (13.4)
Highest educational level	
No education	2015 (41.0)
Primary	883 (18.0)
Secondary	1462 (29.7)
Higher	559 (11.4)
Household’s wealth index	
Poorest	1034 (21.0)
Poorer	1010 (20.5)
Middle	1046 (21.3)
Richer	1019 (20.7)
Richest	810 (16.5)
Currently working	
No	1916 (39.0)
Yes	3003 (61.0)
Standardised attitude to violence SWPER score	0.65 (0.18,0.71)
Standardised social independence SWPER score	− 0.37 (− 0.84,0.23)
Standardised decision making SWPER score	− 0.73 (− 1.50,0.02)

*SWPER* survey-based women’s empowerment index

**Fig. 1:**
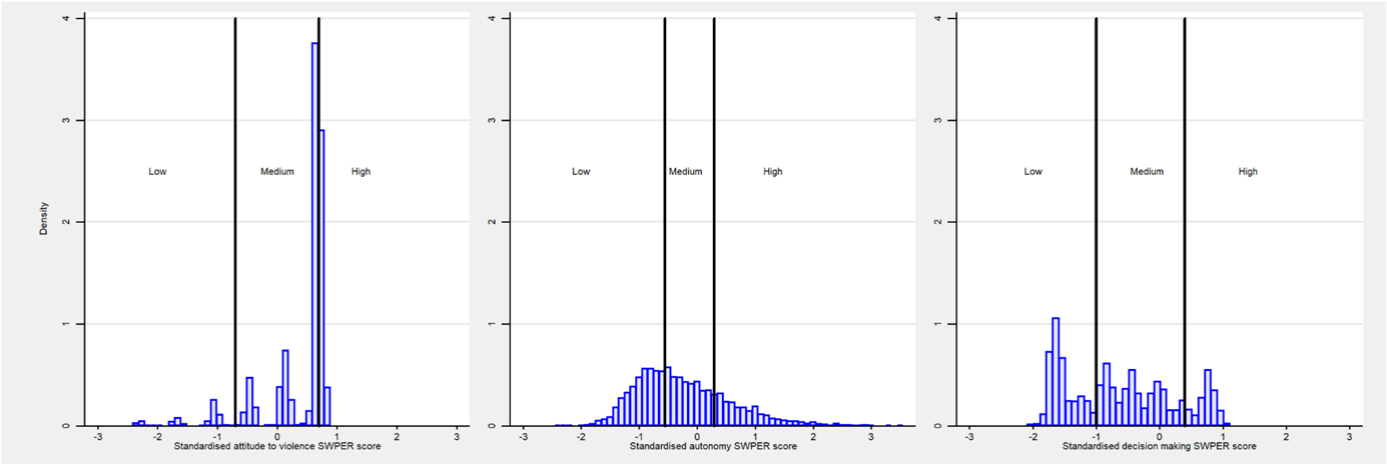
Distribution of the attitude to violence, social independence and decision making SWPER scores with the black lines indicating the cut-offs for the empowerment levels divided in three groups (low, medium and high empowerment)

Using the SWPER index, the standardised score for the attitude to violence domain was 0.65 (0.18, 0.71), for social independence domain it was − 0.37 (− 0.84, 0.23), and for decision making domain it was − 0.73 (− 1.50, 0.02), suggesting a higher level of empowerment in the attitude to violence domain and comparatively lower levels in social independence and decision-making domains (Table 1). Figure 1 shows the distribution of SWPER scores for the ‘attitude to violence,’ ‘social independence,’ and ‘decision making’ domains. Solid lines indicate the cut-offs used to classify empowerment levels into three categories: low, medium, and high. Majority of women (71.8%) were classified as having high empowerment in the ‘attitude to violence’ domain. However, considerably fewer were deemed highly empowered in the ‘social independence’ (23.1%) and ‘decision-making’ (13.6%) domains.

Table 2 presents the levels of women’s empowerment according to various sociodemographic characteristics. Women residing in rural areas tended to have lower levels of empowerment in ‘social independence’ and ‘decision making’ domains compared to those living in urban areas. All provinces except Madhesh had similar distributions for empowerment in the three domains. The province of Madhesh exhibited the lowest level of women empowerment, with only 6.0% of women demonstrating a high level of empowerment in the ‘social independence’ domain and 35.7% reporting low level of empowerment in the ‘attitude to violence’ domain. The Karnali and Sudurpashchim provinces also showed lower percentages of women with high empowerment across the board. Women with no or only primary education exhibited lower empowerment levels across all domains, whereas higher education was prominently linked to high levels in the ‘social independence’ domain. Women from wealthier households, particularly those in the richest quintile, showed higher levels of empowerment across all domains. Also, women who were currently working were more likely to exhibit high empowerment, especially in the ‘decision-making’ domain (Table 2).

**Table 2:** Level of women empowerment by various sociodemographic characteristics

	Level of empowerment								
	Attitude to violence			Social independence			Decision-making		
	Low	Medium	High	Low	Medium	High	Low	Medium	High
Overall	305 (6.3)	1075 (21.9)	3530 (71.8)	1978 (40.2)	1798 (36.6)	1134 (23.1)	1821 (37.0)	2420 (49.2)	669 (13.6)
Age groups									
15–29 years	125 (41.0)	521 (48.5)	1558 (44.1)	666 (33.7)	938 (52.2)	600 (52.9)	1097 (60.2)	893 (36.9)	214 (32.0)
30–39 years	110 (36.1)	351 (32.7)	1144 (32.4)	729 (36.9)	512 (28.5)	364 (32.1)	424 (23.3)	898 (37.1)	283 (42.3)
40–49 years	70 (23.0)	203 (18.9)	828 (23.5)	583 (29.5)	348 (19.4)	170 (15.0)	300 (16.5)	629 (26.0)	172 (25.7)
Area of residence									
Urban	189 (62.0)	673 (62.6)	2251 (63.8)	1144 (57.8)	1146 (63.7)	823 (72.6)	1050 (57.7)	1616 (66.8)	447 (66.8)
Rural	116 (38.0)	402 (37.4)	1279 (36.2)	834 (42.2)	652 (36.3)	311 (27.4)	771 (42.3)	804 (33.2)	222 (33.2)
Province									
Koshi	35 (11.5)	136 (12.7)	518 (14.7)	181 (9.2)	267 (14.8)	241 (21.3)	171 (9.4)	420 (17.4)	98 (14.6)
Madhesh	109 (35.7)	191 (17.8)	589 (16.7)	548 (27.7)	273 (15.2)	68 (6.0)	408 (22.4)	418 (17.3)	63 (9.4)
Bagmati	31 (10.2)	135 (12.6)	423 (12.0)	181 (9.2)	193 (10.7)	215 (19.0)	138 (7.6)	353 (14.6)	98 (14.6)
Gandaki	13 (4.3)	117 (10.9)	480 (13.6)	178 (9.0)	257 (14.3)	175 (15.4)	185 (10.2)	289 (11.9)	136 (20.3)
Lumbini	45 (14.8)	157 (14.6)	566 (16.0)	283 (14.3)	302 (16.8)	183 (16.1)	317 (17.4)	329 (13.6)	122 (18.2)
Karnali	29 (9.5)	167 (15.5)	513 (14.5)	314 (15.9)	262 (14.6)	133 (11.7)	330 (18.1)	294 (12.1)	85 (12.7)
Sudurpashchim	43 (14.1)	172 (16.0)	441 (12.5)	293 (14.8)	244 (13.6)	119 (10.5)	272 (14.9)	317 (13.1)	67 (10.0)
Highest educational level									
No education	163 (53.4)	425 (39.5)	1421 (40.3)	1404 (71.0)	520 (28.9)	85 (7.5)	718 (39.4)	1013 (41.9)	278 (41.6)
Primary	70 (23.0)	215 (20.0)	596 (16.9)	429 (21.7)	367 (20.4)	85 (7.5)	327 (18.0)	426 (17.6)	128 (19.1)
Secondary	62 (20.3)	356 (33.1)	1,043 (29.5)	144 (7.3)	809 (45.0)	508 (44.8)	601 (33.0)	669 (27.6)	191 (28.6)
Higher	10 (3.3)	79 (7.3)	470 (13.3)	< 5 (0.1)	102 (5.7)	456 (40.2)	175 (9.6)	312 (12.9)	72 (10.8)
Household’s wealth index									
Poorest	41 (13.4)	232 (21.6)	757 (21.4)	507 (25.6)	385 (21.4)	138 (12.2)	428 (23.5)	456 (18.8)	146 (21.8)
Poorer	63 (20.7)	238 (22.1)	709 (20.1)	439 (22.2)	397 (22.1)	174 (15.3)	396 (21.7)	490 (20.2)	124 (18.5)
Middle	104 (34.1)	246 (22.9)	695 (19.7)	470 (23.8)	403 (22.4)	172 (15.2)	422 (23.2)	495 (20.5)	128 (19.1)
Richer	56 (18.4)	220 (20.5)	741 (21.0)	391 (19.8)	375 (20.9)	251 (22.1)	378 (20.8)	498 (20.6)	141 (21.1)
Richest	41 (13.4)	139 (12.9)	628 (17.8)	171 (8.6)	238 (13.2)	399 (35.2)	197 (10.8)	481 (19.9)	130 (19.4)
Currently working									
No	145 (47.5)	429 (39.9)	1341 (38.0)	773 (39.1)	681 (37.9)	461 (40.7)	838 (46.0)	874 (36.1)	203 (30.3)
Yes	160 (52.5)	646 (60.1)	2189 (62.0)	1205 (60.9)	1117 (62.1)	673 (59.3)	983 (54.0)	1546 (63.9)	466 (69.7)

Table 3 shows the prevalence of hypertension and the proportions of those aware of hypertension and treated for hypertension. In our sample, the overall prevalence of hypertension was 11.5%. Hypertension was particularly prevalent among older women, for example, hypertension prevalence was 4.7% and 23.8% in the age groups of 15–29 years and 40–49 years, respectively. Women living in urban areas had higher prevalence of hypertension than those living in rural areas (12.3% vs. 10.2%). Women with lower educational level and from wealthier households were more likely to have hypertension than their counterparts. We found significant differences in the prevalence of hypertension according to levels of empowerment in the ‘attitude to violence’, ‘social independence’, and ‘decision-making’ domains of the SWPER index.

**Table 3:** Hypertension prevalence, awareness of hypertension, and hypertension treatment by various sociodemographic factors and SWPER domains

	Hypertension prevalence (N = 4,919)		Awareness of hypertension (N = 568)			Hypertension treatment (N = 568)
	Yes	p-value	Yes	p-value	Yes	p-value
Overall	568 (11.5)		199 (35.0)		82 (14.4)	
Age groups		< 0.001		0.006		0.016
15–29 years	104 (4.7)		25 (24.0)		7 (6.7)	
30–39 years	201 (12.5)		66 (32.8)		27 (13.4)	
40–49 years	263 (23.8)		108 (41.1)		48 (18.3)	
Area of residence		0.027		0.075		0.054
Urban	384 (12.3)		144 (37.5)		63 (16.4)	
Rural	184 (10.2)		55 (29.9)		19 (10.3)	
Province		< 0.001		0.12		0.056
Koshi	83 (12.0)		38 (45.8)		16 (19.3)	
Madhesh	67 (7.5)		28 (41.8)		15 (22.4)	
Bagmati	105 (17.8)		37 (35.2)		15 (14.3)	
Gandaki	103 (16.9)		35 (34.0)		16 (15.5)	
Lumbini	110 (14.2)		30 (27.3)		9 (8.2)	
Karnali	62 (8.7)		17 (27.4)		4 (6.5)	
Sudurpashchim	38 (5.8)		14 (36.8)		7 (18.4)	
Highest educational level		0.011		0.56		0.85
No education	261 (13.0)		85 (32.6)		40 (15.3)	
Primary	110 (12.5)		42 (38.2)		13 (11.8)	
Secondary	141 (9.6)		49 (34.8)		21 (14.9)	
Higher	56 (10.0)		23 (41.1)		8 (14.3)	
Household’s wealth index		< 0.001		< 0.001		< 0.001
Poorest	91 (8.8)		22 (24.2)		6 (6.6)	
Poorer	114 (11.3)		28 (24.6)		9 (7.9)	
Middle	115 (11.0)		37 (32.2)		10 (8.7)	
Richer	101 (9.9)		40 (39.6)		16 (15.8)	
Richest	147 (18.1)		72 (49.0)		41 (27.9)	
Currently working		0.14		0.005		0.005
No	205 (10.7)		87 (42.4)		41 (20.0)	
Yes	363 (12.1)		112 (30.9)		41 (11.3)	
Attitude to violence		0.045		0.56		0.22
Low	27 (8.9)		12 (44.4)		6 (22.2)	
Medium	108 (10.0)		38 (35.2)		11 (10.2)	
High	432 (12.2)		148 (34.3)		65 (15.0)	
Social independence		0.002		0.26		0.20
Low	261 (13.2)		90 (34.5)		43 (16.5)	
Medium	172 (9.6)		54 (31.4)		18 (10.5)	
High	134 (11.7)		54 (40.3)		21 (15.7)	
Decision-making		< 0.001		0.17		0.29
Low	147 (8.1)		42 (28.6)		20 (13.6)	
Medium	317 (13.1)		117 (36.9)		42 (13.2)	
High	103 (15.4)		39 (37.9)		20 (19.4)	

Of the women diagnosed with hypertension, 35.0% were aware of their condition, and 14.4% were taking medication for hypertension control. Awareness of hypertension were more prevalent among older women (24.0% in 15–29 years vs. 41.1% in 40–49 years), those residing in urban areas (37.5% vs. 29.9% in rural areas), those with higher education (41.1% vs. 32.6% among women with no education), and those from the richest households (49.0% vs. 24.2% in poorest households) (Table 3). Interestingly, women who were currently working were less likely to be aware of their hypertension compared to those who were not working (30.9% vs. 42.4%). Only 14.4% of those having hypertension was being treated for their hypertension. Treatment of hypertension were more prevalent among older women (6.7% in 15–29 years vs. 18.3% in 40–49 years), those residing in urban areas (16.4% vs. 10.3% in rural areas), and those from the richest households (27.9% vs. 6.6% in poorest households). Interestingly, no significant differences in the proportion of hypertension awareness and treatment were observed based on the levels of ‘attitude to violence’, ‘social independence’, and ‘decision-making’ domains of the SWPER index (Table 3).

**Fig. 2:**
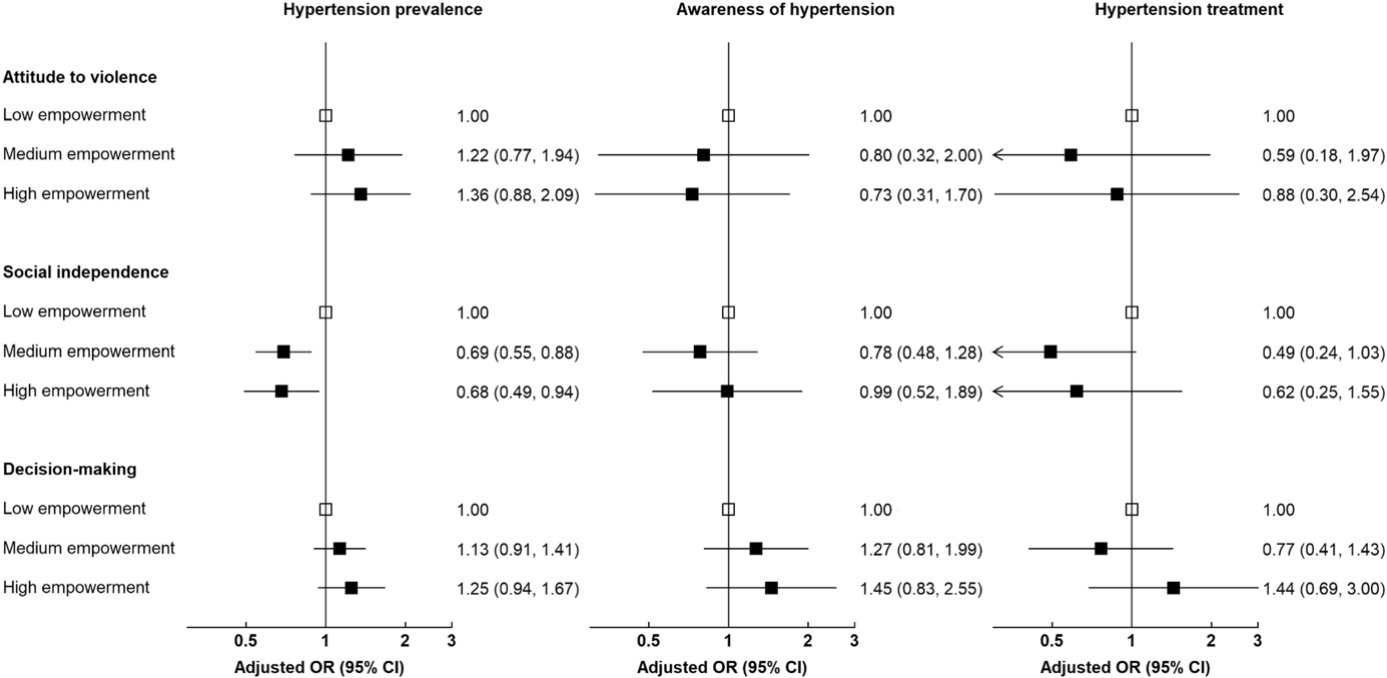
Associations of women empowerment domains with hypertension prevalence, awareness and treatment of hypertension. Multiple logistic regressions were adjusted for age, province, area of residence, education level, employment status, and household wealth index. Odds ratios (ORs) are represented by squares, and their corresponding 95% CIs are represented by lines

The results from the multiple logistic regression with adjustment for sociodemographic factors showed a significant association of medium and high level of empowerment in the ‘social independence’ domain and lower odds of hypertension, with aOR = 0.69 (95% CI 0.55–0.88) and aOR = 0.68 (95% CI 0.49–0.94), respectively (Fig. 2). We observed no significant association between hypertension prevalence and high empowerment in other domains of the SWPER index. However, there were no statistically significant associations between empowerment levels in the domains of women empowerment and awareness and treatment of hypertension.

## Discussion

To the best of our knowledge, our study is the first study utilising nationally representative data to investigate the associations between different aspects of women empowerment (as estimated by the SWPER index) and the prevalence, awareness, and treatment of hypertension among reproductive-aged women (15–49 years) in Nepal. We found that the overall prevalence of hypertension was 11.5% and among those with hypertension, 35.0% were aware of their condition and 14.4% were taking medication to control hypertension. Our study further revealed that compared to women with low level of empowerment in the ‘social independence’ domain, those with medium and high level of empowerment were less likely to suffer from hypertension. However, other aspects of women empowerment (e.g., ‘attitude to violence’ and ‘decision making’) were not associated with hypertension prevalence, awareness, and treatment.

Nepal has made notable progress in advancing women’s rights and achieving gender-related SDG commitments, with 91.7% of the required legal frameworks established to promote, enforce, and monitor gender equality, specifically addressing violence against women [18]. Our study revealed that a significant majority, approximately 71.8%, of women, displayed high empowerment in the ‘attitude to violence’ domain. However, the level of empowerment in the ‘social independence’ and ‘decision-making’ domains was considerably lower, with only 23.1% and 13.6% of women achieving high empowerment, respectively. Furthermore, we observed substantial variations in women’s empowerment across the ‘attitude to violence’, ‘social independence’, and ‘decision-making’ domains based on the province in which they resided. These regional variations imply that socio-cultural factors unique to each province could significantly influence women’s empowerment levels. Hence, fulfilling Nepal’s gender-related SDG commitments requires continued dedication. To expedite this process, the Government of Nepal, in collaboration with the Delegation of the European Union in Nepal and the United Nations, has launched a four-year joint program, “Empowered Women, Prosperous Nepal” [23]. This initiative seeks to further gender equality and empower women and girls, guaranteeing equal access to economic, labour, and social rights for everyone, irrespective of their gender or diversity [23].

Our study offers several important insights into the prevalence and management of hypertension among Nepalese women. Our findings reveal that 11.5% of women aged 15 to 49 years have hypertension, with prevalence rates rising from a modest 4.7% in the 15–29 years age group to a substantial 23.8% in the 40–49 years age group. This pronounced increase in hypertension prevalence with age corresponds with previous study findings [3–6]. Moreover, we observed distinct disparities in hypertension prevalence was related to area of residence and household socio-economic status, mirroring results from other studies [5, 6]. Hypertension prevalence among Nepalese women was comparable to hypertension prevalence among women in other south Asian countries [24]. Alarmingly, we found that a large proportion of hypertensive women were either unaware of their condition or not receiving the necessary antihypertensive treatment, thus indicating a pressing need for enhanced awareness, screening, and management strategies. Similar rates of hypertension awareness and treatment were reported in prior studies [3–6]. For example, one recent study, based on a pooled sample of 9682 participants from two consecutive STEPwise approach to Surveillance (STEPS) surveys conducted in Nepal in 2013 and 2019, reported that the prevalence of hypertension awareness was 20%, while hypertension treatment prevalence was 10.3% [6]. These findings highlight the necessity of exploring socio-economic factors and health-seeking behaviours that could potentially influence hypertension awareness and treatment among Nepalese women. Additionally, the higher prevalence of hypertension among working women, combined with lower awareness and treatment rates, may be influenced by time constraints and the competing demands of work and household responsibilities [25]. These factors, along with workplace stress, could hinder their ability to prioritise healthcare, warranting further investigation.

Our study highlights a statistically significant association between high empowerment in the ‘social independence’ domain of the SWPER index and a decreased likelihood of hypertension prevalence. This is in line with broader research linking women empowerment to health outcomes. Previous studies, for example, have established a strong correlation between high levels of social independence and utilisation of maternal health services [26]. Another comprehensive study analysing 50 Demographic Health Surveys found that children of women empowered in the social independence domain were more likely to receive childhood vaccinations [12]. Upon examining the variables composing the ‘social independence’ domain—such as reading frequency, women’s education, age at first birth, age at first cohabitation, and the differences in age and education between the woman and her partner—the reasoning behind this association becomes evident [12, 16]. The ‘social independence’ domain essentially reflects a woman’s awareness and social status, both likely to influence her understanding of the significance of healthy lifestyles and her ability to make informed health decisions [26]. Women with greater social independence are likely to have a better understanding of hypertension risk factors and the significance of preventive health behaviours. Furthermore, such independence may improve women’s access to health-related resources, enabling them to seek appropriate healthcare advice and hypertension treatment. More focus should also be given to factors such as harmful gender norms, which often restrict women’s physical activity, a key determinant of hypertension and other NCDs. Thus, our findings stress the critical role of women empowerment, particularly social independence, in the policy and programmatic interventions targeting NCDs, such as hypertension in Nepal and in similar settings (for example, LMICs) undergoing a transition from communicable to non-communicable diseases.

Our study notably contributes to the existing body of literature by looking into the association between women empowerment and the prevalence, awareness, and treatment of hypertension in Nepal. Utilising standardised methodologies and questionnaires, as well as the SWPER index [15, 16]—a globally recognised and validated measure of women empowerment—our research findings exhibit substantial reliability. Furthermore, we used a nationally representative NDHS dataset with measured blood pressure information. Despite these strengths, our study also has several limitations. The SWPER index, despite its robustness, does not capture all dimensions of women empowerment—a multifaceted construct that extends into economic, sociocultural, familial, interpersonal, legal, political, societal, and psychosocial dimensions [12, 26]. Furthermore, SWPER index does not include information on personal asset ownership, economic participation, and participation in governance processes [15]. Our study included only married or cohabitating women and therefore our findings should not be generalised to all women. Information on awareness and treatment of hypertension was self-reported and not cross-validated against medical records which potentially can introduce reporting bias. Despite adjustments for a variety of sociodemographic factors, there are possibilities of residual confounding in the associations of women empowerment domains and hypertension prevalence, awareness, and treatment.

## Conclusions

This study reported low levels of awareness and treatment of hypertension among women in Nepal. Furthermore, it underscores a significant association between women empowerment, particularly in the domain of social independence, and the prevalence of hypertension. These findings highlight the critical need to promote women empowerment as an integral component of strategies to combat non-communicable diseases, including hypertension, in Nepal and other LMICs. It is essential to undertake further research that incorporates a more comprehensive range of empowerment indicators and includes diverse groups of women to deepen our understanding and to guide the development of gender-sensitive health policies and interventions.

## Supplementary Information

Below is the link to the electronic supplementary material.

Supplementary file1 (DOCX 16 kb)

## Data Availability

The 2016 Nepal Demographic and Health Survey dataset used in this study are publicly available at this link: https://dhsprogram.com/data/
