# Supplementary material for: Women empowerment and hypertension in Nepal: a nationally representative survey analysis
Source: J Public Health Policy. 2025 Aug 13;46(4):778–94. doi: 10.1057/s41271-025-00593-7 (PMC12586156; doi:10.1057/s41271-025-00593-7)
Supplement: Supplementary file 1 — Supplementary file1 (DOCX 16 kb) [file 41271_2025_593_MOESM1_ESM.docx]

**Women empowerment and hypertension in Nepal:**

**a nationally representative survey analysis**

Md Shajedur Rahman Shawon^1*^, Mohammad Rifat Rahman^2^, Tanij Fahima^3^, Ferdousy Jannat^4^, Fariha Binte Hossain^5^

^1^ Centre for Big Data Research in Health, University of New South Wales, Sydney, Australia

^2^ Department of Banking & Insurance, Faculty of Business Administration, University of Chittagong, Chattogram, Bangladesh

^3^ Mukti Cox'sBazar, Cox'sBazar, Bangladesh

^4^ Independent University Bangladesh, Dhaka, Bangladesh

^5^ School of Population Health, University of New South Wales, Sydney, Australia

**Supplementary materials**

**Table S1: Items used in the development of the survey-based women’s empowerment index**

| **Survey items** | **Code or unit** |
| --- | --- |
| Beating not justified if wife goes out without telling husband | Justified=–1; don’t know=0; not justified =1 |
| Beating not justified if wife neglects the children | Justified=–1; don’t know=0; not justified =1 |
| Beating not justified if wife argues with husband | Justified=–1; don’t know=0; not justified =1 |
| Beating not justified if wife refuses to have sex with husband | Justified=–1; don’t know=0; not justified =1 |
| Beating not justified if wife burns the food | Justified=–1; don’t know=0; not justified =1 |
| Frequency of reading newspaper or magazine | Not at all=0; <once a week=1; ≥once a week=2 |
| Woman’s education in completed years of schooling | Years |
| Education difference: woman’s minus husband’s completed years of schooling | Years |
| Age difference: woman’s age minus husband’s age | Years |
| Age at first cohabitation | Years |
| Age of woman at first birth* | Years |
| Who usually decides on respondent’s health care | Husband or other alone=–1; joint=0; respondent alone=1 |
| Who usually decides on large household purchases | Husband or other alone=–1; joint=0; respondent alone=1 |
| Who usually decides on visits to family or relatives | Husband or other alone=–1; joint=0; respondent alone=1 |
| * Imputed for women who had not had a child.  Adapted from: Ewerling F, Lynch JW, Victora CG, van Eerdewijk A, Tyszler M, Barros AJD. The SWPER index for women's empowerment in Africa: development and validation of an index based on survey data. Lancet Glob Health. 2017 Sep;5(9):e916-e923. doi: 10.1016/S2214-109X(17)30292-9. Epub 2017 Jul 26. PMID: 28755895; PMCID: PMC5554795. | |
